# Effect of calcitriol supplementation on infectious biomarkers in patients with positive systemic inflammatory response: A Randomized Controlled Trial

**DOI:** 10.1097/MS9.0000000000001643

**Published:** 2024-01-03

**Authors:** Mohammad Sistanizad, Sara Salarian, Mehran Kouchek, Seyedpouzhia Shojaei, MirMohammad Miri, Farnoosh Masbough

**Affiliations:** a Department of Clinical Pharmacy, School of Pharmacy; b Prevention of Cardiovascular Disease Research Center; c Department of Critical Care Medicine, Imam Hossein Medical and Educational Center, Shahid Beheshti University of Medical Sciences, Tehran, Iran

**Keywords:** biomarker, calcitriol, PCT, presepsin, sepsis, SIRS

## Abstract

**Background:**

Sepsis is one of the common causes of hospitalization of patients in intensive care units. A significant role for vitamin D in sepsis has been proposed, which is due to its active metabolite, calcitriol.

**Aims:**

Evaluate the effect of calcitriol supplementation on infectious biomarkers, including procalcitonin and presepsin.

**Methods:**

Patients with sepsis were divided into intervention and control group. Patients in the intervention group received intravenous calcitriol daily for 3 days. The serum levels of procalcitonin and presepsin were evaluated on days 0, 3, and 5 after administration.

**Results:**

Fifty-two SIRS-positive patients were evaluated. Baseline characteristics, changes in Sequential Organ Failure Assessment (SOFA) score and blood levels of vitamin D were not significantly different between the two groups. Procalcitonin levels on day 5 and the differences between day 5 and 0 were significantly lower in the intervention group (*P* = 0.02). Presepsin on the third and fifth days in the intervention group was reduced, but in the control group, there was an ascending trend. However, there was no significant difference between the two groups on days 3 and 5 (*P* = 0.17 and *P* = 0.06, respectively) or between days 3 as well as 5 and the baseline presepsin level (*P* = 0.93 and *P* = 0.92, respectively). The ICU length of stay and 28-day mortality did not differ significantly either between the two arms of the study.

**Conclusions:**

Finally, the results of this study showed that the administration of intravenous calcitriol could reduce the levels of procalcitonin but did not have a significant effect on presepsin.

## Introduction

Highlights

- A unique feature of the current study is the use of the active intravenous form of vitamin D rather than nutritional vitamin D3.
- Intravenous calcitriol could reduce the levels of procalcitonin as a diagnostic marker for sepsis but did not have a significant effect on presepsin.
- The level of procalcitonin on day 5 after calcitriol injection, as well as the difference in the level of day 5 with the baseline in the intervention group, was significantly different from the control group.

Sepsis and systemic inflammatory response syndrome (SIRS) are common findings in critically ill patients^1^. SIRS due to infective causes has been defined as sepsis in recent sepsis guidelines^2^. Differentiating sepsis from SIRS is crucial, as antimicrobial consumption and resistance could increase if antibiotics were started in patients with non-infectious causes. On the other hand, late initiation of antimicrobials in septic patients could significantly augment the mortality rate^3,4^. Recent studies have focused on novel biomarkers to accelerate the diagnosis of sepsis and differentiate it from SIRS^5,6^.

C-reactive protein and procalcitonin are important markers for the diagnosis of sepsis^7^. Compared to CRP, procalcitonin is more specific for bacterial infection, but both markers have a limited ability to distinguish sepsis from SIRS^8^. In 2005, a new biomarker, presepsin, was identified. Presepsin is a soluble N-terminal fragment of the cluster of differentiation (CD) marker protein CD14, which is released into circulation during infection. CD14 is a surface marker of glycoprotein which is present on the surface of monocytes and macrophages and is a specific receptor for bacterial lipo-polysaccharides (LPS). When LPS binds to CD14, the toll-like receptor-4 (TLR4) is activated, and a host inflammatory response against the infectious agent follows. Circulating proteases activate a part of CD14 and form a 64-amino acid chain called presepsin^9^, which has better sensitivity and specificity in the diagnosis of sepsis than procalcitonin or CRP^10^.

Because of the involvement of the immune system in the process of SIRS, several studies have evaluated the role of different interventions in modulating the immune system in order to decrease the consequences of SIRS in critically ill patients^11–13^. Recently, one such intervention is vitamin D, which affects several interleukins and tumour necrotizing factors (TNF).

Being fat-soluble, vitamin D affects the immune system with its active metabolite 1,25-dihydroxy vitamin D3 (1, 25 (OH_2_) D), or calcitriol^14^. In addition to its involvement in known functions, including calcium and bone homoeostasis, recent studies have shown vitamin D to have non-skeletal biological effects, such as antimicrobial and immunomodulatory effects^15^. Calcitriol promotes the signalling of innate immunity, including macrophages, monocytes, and epithelial cells, to increase the production of IL-37 and cathelicidin, a relevant antimicrobial peptide. In general, calcitriol improves both innate and adaptive immune systems by producing anti-inflammatory cytokines, that is IL-10, and inhibiting pro-inflammatory cytokines such as IL-2, IL-8, and TNFα^16–18^.

Vitamin D deficiency is common in the general population as well as critically ill patients^19^. Several studies have shown that a low plasma level of vitamin D is related to increased susceptibility to infections, increased duration of mechanical ventilation^20,21^, and increased mortality in hospitalized patients^22–24^. Nonetheless, other studies have not found any relation between vitamin D administration and decreased morbidity and mortality^23,24^.

Quraishi *et al.* ^25^ showed that the administration of high dose vitamin D in severe sepsis and septic shock significantly influenced cytokine expression and reduction in systemic levels of IL-1β and IL-6. Leaf *et al.* ^26^ showed that a single intravenous dose of 2 µg calcitriol in patients with severe sepsis and septic shock significantly increased levels of anti-inflammatory cytokines like IL-10.

The current study is an open-labelled randomized controlled trial (RCT) conducted among critically ill patients to evaluate the effect of calcitriol supplementation on infectious biomarkers, including procalcitonin and presepsin. To the best of our knowledge, this is the first study to evaluate the role of an active form of vitamin D on presepsin as a surrogate of sepsis in SIRS-positive patients.

## Materials and methods

### Methods

This open-label RCT was conducted at the 30-bed medical-surgical intensive care unit of a teaching. The ICU team, comprising intensivists, clinical pharmacy specialists, and nurses, visits patients daily.

### Patients

#### Inclusion and exclusion criteria

All adult ICU patients who met the SIRS criteria and were candidates for starting antibiotics based on the evaluation of the ICU team were assessed for inclusion in the study. Patients who met two of the following four criteria were defined as SIRS-positive^27^:

1. Temperature greater than 38°C or less than 36°C.
2. Heart rate greater than 90 beats/min.
3. Respiratory rate greater thanr30 breaths/min or arterial CO_2_ less than 32 mm Hg.
4. White blood cell count greater than 12 000 or less than 4000 cells/ml or greater than 10% band forms.

Any patient meeting the following criteria were omitted from the study:

1. Serum calcium greater than or equal to 10 mg/dl or phosphate greater than or equal to 6 mg/dl within the previous 48 hours.
2. Current or recent therapy with vitamin D at doses greater than 1000 IU per day, or calcitriol at any dose within the previous 7 days.
3. History of sarcoidosis, metabolic bone disease, or end-stage renal disease.
4. Pregnancy or lactation.
5. Expected to die or leave the ICU within 48 h.
6. Receiving effective antibiotic for more than 48 h before recruitment.

### Study design and sample collection

In this prospective RCT, eligible patients, based on the defined inclusion and exclusion criteria, were randomly assigned to intervention and control groups after written consent was provided by the patient or their attorney. Randomization was performed by a simple randomization method using numbers generated randomly by Excel software. All patients were managed based on ICU protocols. In the intervention group, in addition to the standard treatment, patients received 1-microgram intravenous calcitriol (DECOSTRIOL, Germany) in 100 ml isotonic saline infused over 30 minutes for three consecutive days.

Baseline demographic information and clinical characteristics, including age, sex, reason for ICU admission, patient temperature, and vital signs of the recruited patients were documented, and blood samples for measuring liver and kidney function, culture, CBC, 25-hydroxyvitamin D3, and procalcitonin blood levels were collected. All recruited patients were evaluated daily, and Sequential Organ Failure Assessment (SOFA) scores were recorded for 5 days. Length of ICU stay and 28-day mortality were also documented.

To measure infection markers, presepsin, and PCT, 5 ml samples of venous blood were collected from each patient at three-time points: immediately before drug administration, 3 days after, and 5 days after calcitriol injection. Serum was separated by centrifugation (4000 rpm for 5 minutes) within 1 h of collection and was stored at −80°C.

### Sample Size

This study is the first to evaluate the effect of calcitriol on presepsin. It was run as a pilot study, and a sample size of 15 was considered for each group.

### Laboratory analyses

Evaluation of vitamin D and procalcitonin levels: Blood levels of PCT were measured at baseline and on the third and fifth days after calcitriol injection. Vitamin D was measured at baseline and on the fifth day of the study by hospital laboratory through chemiluminescence immunoassay using a device from Centor .

Evaluation of presepsin levels: The human blood ELISA kit (ZellBio, Germany) was used to evaluate the blood levels of presepsin using Biotin double antibody sandwich technology according to the manufacturer’s structures.

### Statistical analysis

All statistical analyses were performed using SPSS for Windows (Version 21.0; SPSS Inc.). Quantitative data were tested for normality of distribution by Kolmogorov–Smirnov test and then compared by two-tailed Unpaired Student’s *t*-test and Mann–Whitney U test for normal and non-normal data, respectively. Qualitative data were analyzed by the χ^2^ or Fisher exact test, and a *P* value of less than 0.05 was considered significant.

## Results

**Figure 1:**
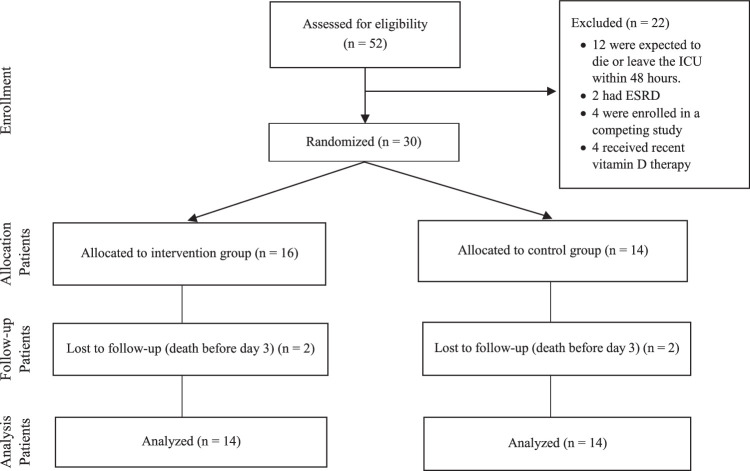
Consort chart of the study^28^.

The current study was performed between September 2017 and May 2018. Fifty-two SIRS-positive patients were evaluated based on the defined inclusion and exclusion criteria, and 30 patients, 16 in the intervention group and 14 in the control group, were recruited. Three patients, 2 in intervention and 1 in control arms of the study, were excluded because of death before day 3. Ultimately, 27 patients (17 men and ten women) were included in the final analysis (Fig. 1). There were no statistically significant differences in baseline demographic and clinical characteristics of the patients in the two arms of the study (Table 1). Physiological variables and laboratory values (temperature, culture, CBC, liver, and kidney functions) did not significantly differ between the two study arms at baseline or during the study.

**Table 1:** Baseline demographic and clinical characteristics

Characteristics	Intervention *(n*=14)	Control *(n*=13)	*P*
Age, year, mean±SD	50.21±23.11	61.69±20.71	0.18
Sex, *n* (%)			0.695
Female	6 (43)	4 (31)	
Male	8 (57)	9 (69)	
Weight, kg, mean±SD	73±6.84	70.16±6.83	0.33
APACHE II	21.36±5.42	17.07±5.79	0.07
1,25 OH-D baseline mean±SD	11.36±5.57	13.95±11.76	0.48
Cause of hospitalization, *n* (%)			0.31
CVA	3 (21)	7 (53)	
MT	5 (35)	3 (23)	
BT	2 (15)	0	
Seizure	1 (7)	1 (8)	
LOC	2 (15)	0	
Peritonitis	1 (7)	1 (8)	
Rectorrhagia	0	1 (8)	

APACHE II, Acute Physiology and Chronic Health Evaluation II; BT, brain tumour; CVA, cardiovascular accident; LOC, loss of consciousness; MT, multiple trauma; PCT, procalcitonin; SOFA, Sequential Organ Failure Assessment.

### Patient status

All patients were connected to a mechanical ventilator and followed the usual hospital diet. Ten patients in the intervention group and 8 in the control group received vasopressor.

### Culture result and antibiotic regimen

Culture samples are related to blood, urine, and sputum. The predominant microorganisms in both groups were *Staphylococcus aureus*, *Acinobacter* and *Pseudomonas aeruginosa*. No significant difference was observed between the two groups in the culture results (*P* = 0.66) or the antibiotics regimen (*P* = 0.96).

### Laboratory Data

#### 1,25-dihydroxy vitamin D

No significant difference in vitamin D levels at baseline or the fifth day of the study was observed between the two groups. After 5 days of calcitriol injections, vitamin D levels increased from 11.37±5.57 to 12.27±5.39 in the intervention group and decreased from 13.96±7.76 to 11.86±7.63 in the control group (*P* = 0.49).

### Procalcitonin

**Figure 2:**
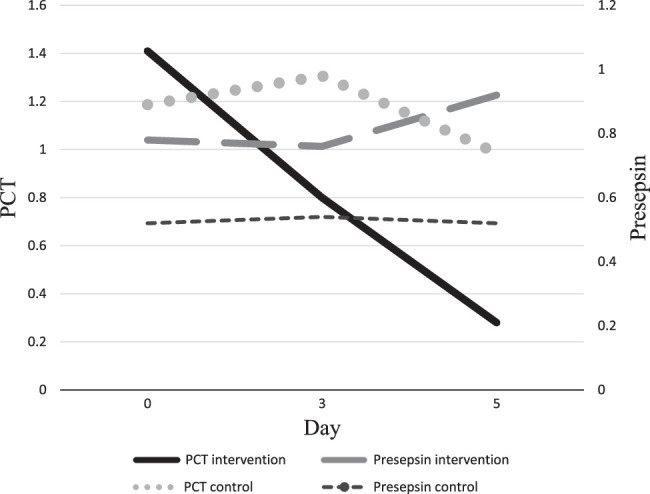
Procalcitonin and presepsin levels in intervention and control group. PCT, procalcitonin.

Procalcitonin levels on the third and fifth days of the study were 0.98±1.25 versus 0.80±0.79 (*P* = 0.85) and 0.74±0.8 versus 0.28±0.35 (*P* = 0.02) in the control and intervention arms of the study, respectively. Analysis of within-group changes in procalcitonin revealed a significant decrease in procalcitonin levels from baseline to the fifth day of the study in the intervention group compared to the control (−1.13±1.33 versus −0.15±0.96, *P* = 0.02). Data are shown in Table 2 and Figure 2.

**Table 2:** Serum procalcitonin and presepsin levels on day 3 and 5 and percent changes during study days in intervention and control group

		Intervention	Control	
	Days	Mean±SD	Mean±SD	*p*
Procalcitonin				
µg/ml				
	Baseline	1.41±1.37	0.89±1.20	0.31
	3	0.80±0.79	0.98±1.25	0.85
	5	0.28±0.35	0.74±0.80	0.02
Changes				
	3-0	-0.61±0.87	0.09±1.13	0.20
	5-0	-1.13±1.33	-0.15±0.96	0.02
Presepsin				
µg/ml				
	Baseline	0.78±0.47	0.52±0.23	0.09
	3	0.76±0.37	0.54±0.33	0.17
	5	0.92±0.47	0.53±0.34	0.07
Changes				
	3-0	-0.02±0.22	0.02±0.09	0.93
	5-0	0.14±0.02	0.01±0.01	0.92

### Presepsin

**Figure 3:**
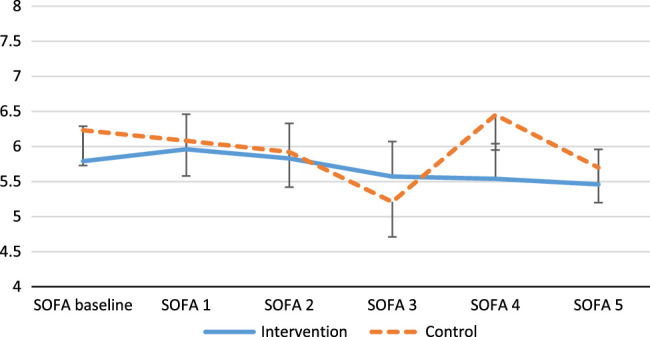
Sequential Organ Failure Assessment (SOFA) score.

Mean serum presepsin levels for the control and intervention groups were 0.54±0.33 and 0.76±0.37 µg/mL (*P* = 0.17), respectively, on day 3 and 0.53±0.34 µg/ml and 0.92±0.47 (*P* = 0.07), respectively, on day 5 of the study. No statistically significant difference was seen the percentage of changes in presepsin levels between the groups on days 3 and 5 of the study (Table 2 and Fig. 3).

### Clinical and safety outcomes

Patients in the both groups had a similar 28-day mortality rate (6 vs. 3, *P* = 0.08), length of ICU stay (8.77± 8.08 vs. 10.69±8.28, *P* = 0.55), and Sequential Organ Failure Assessment (SOFA) score at baseline and on days 1–5 (Fig. 3). No patient developed hypercalcemia within 72 h of drug administration.

### 28-day mortality

Although the 28-day mortality rate in the intervention group was lower, the difference was not statistically significant (*P* = 0.083).

## Discussion

In this open-labelled randomized controlled study, the effects of intravenous calcitriol on biomarkers of infection, such as procalcitonin and presepsin, were evaluated. Our main finding was a significant decrease in PCT levels in the intervention group on the fifth day of the study.

Several studies have investigated the association between vitamin D and sepsis. Low levels of vitamin D reduce the production of cathelicidin by macrophages infected with Mycobacterium tuberculosis, while in-vitro treatment with vitamin D increases the production of cathelicidin and improves the killing of microorganisms^29^.

The link between vitamin D and sepsis is not limited to cathelicidin and tuberculosis. Cathelicidin has anti-inflammatory effects against a wide range of Gram-positive, Gram-negative bacteria^30,31^ and some viruses and fungi^32,33^. Moreover, vitamin D has other anti-inflammatory effects that are not related to cathelicidin, including the reduction of pro-inflammatory cytokines and increased levels of anti-inflammatory cytokines^16,34^.

In a double-blind clinical trial on 67 critically ill patients with severe sepsis and septic shock, patients receiving a single dose of 2 µg/ml calcitriol compared with patients receiving a placebo showed increased cathelicidin production and mRNA expression, consistent with the in-vitro effects of vitamin D^35^.

The results of another study on sepsis, conducted in 2014 by Megan, showed that vitamin D deficiency within 30 days of hospitalization in patients with severe sepsis or septic shock significantly increased 30-day mortality. Furthermore, mortality may be reduced by adequate concentrations of vitamin D supplementation at 30 days after admission^36^.

A dose of 1 µg was chosen for this study based on multiple randomized controlled trials of calcitriol in patients with chronic kidney disease. Additionally, another randomized controlled trial study evaluating calcitriol in severe sepsis used a dose of 2 µg, and no adverse effect was reported^26^.

In the current study patients receiving an active form of vitamin D had a lower mortality rate than the control group but this difference was not statistically significant (*P* value = 0.083).

Zhaoyan examined the association between vitamin D levels in 236 patients with sepsis in the ICU and mortality and procalcitonin levels and reported that vitamin D levels had a negative linear correlation (r = -0.78) with procalcitonin levels, indicating a significant relationship between low vitamin D levels and increased PCT (*P* < 0.01)^37^. In the present study, similar results were obtained; the level of procalcitonin on day 5 after calcitriol injection as well as the difference in levels between baseline and day 5 in the intervention group were significantly different from the control group. There was no significant difference in vitamin D levels in the two study groups; however, on day 5, the intervention group had higher levels than the control group. Considering the lab measures 25(OH)-Vit D levels, and this form of vitamin D is one step before its active form, calcitriol, the precise level of vitamin D cannot be determined. A unique feature of the current study is the use of the active intravenous form of vitamin D rather than nutritional vitamin D3.

There is very little information available today about the kinetics of presepsin, when it reaches a fixed level and when it fluctuates. In a study by Fahmy (2014), levels of presepsin were significantly higher in SIRS-positive patients than in the healthy group. In sepsis, the highest concentration of this biomarker was detected on day 3 after diagnosis and had a decreasing trend until day 7. Septic patients with reduced levels of presepsin on day 7 were most likely to have improved clinical status. These findings suggest that measuring presepsin levels may help monitor the effectiveness of treatment^38^.

Although the current study found no significant change in the levels of presepsin in the two groups, the mean level of presepsin on day 3 was lower in the intervention group, but it did not decrease in the control group.

We acknowledge the limitations of this study, including the low sample size and the impossibility of homogenizing the two groups in terms of the drug regimens. It must be noted that another limitation in this study is the single-centre design. Additional research with a longer follow-up is advised.

## Conclusion

In conclusion, the findings of the current study showed that intravenous calcitriol administration may decrease plasma levels of procalcitonin as a diagnostic biomarker for sepsis, but it does not significantly affect presepsin.

## Ethical approval

The current study was approved by the ethics committee of Shahid Beheshti University of Medical Sciences (IR.SBMU.PHNM.1396.885) and is registered in the Iranian Registry of Clinical Trials (IRCT20180522039N1).

## Consent

I, Farnoosh Masbough hereby declare that I participated in the study and in the development of the manuscript titled “Effect of calcitriol supplementation on infectious biomarkers in patients with positive systemic inflammatory response: A Randomized controlled Trial “. I have read the final version and give my consent for the article to be published in Annals of Medicine and Surgery. Thank you for your support. Sincerely, Farnoosh Masbough The Patent consent is attached at the bottom of this file.

## Sources of funding

This research did not receive any specific grant from funding agencies in the public, commercial, or not-for-profit sectors.

## Author contributions

M.S.: study design, data collection, data analysis, conducting the study, and drafting the manuscript; M.K.: study design, conducting the study, and supervising the study; M.M.M., S.P.S., and S.S.: study design and conducting the study; F.M.: data collection, statistical analysis, and writing the final manuscript.

## Conflicts of interest disclosure

The authors declared that there is no conflict of interest.

## Research registration unique identifying number (UIN)

The current study was approved by the ethics committee of Shahid Beheshti University of Medical Sciences (IR.SBMU.PHNM.1396.885) and is registered in the Iranian Registry of Clinical Trials (IRCT20180522039N1). Informed consent was obtained from each patient’s next of kin before they were recruited into the study. Registration date: 2018-10-07, 1397/07/15.

## Guarantor

Farnoosh Masbough Mohammad Sistanizad.

## Data availability statement

Data from this study are publicly available.

## Provenance and peer review

Not commissioned, externally peer-reviewed.
